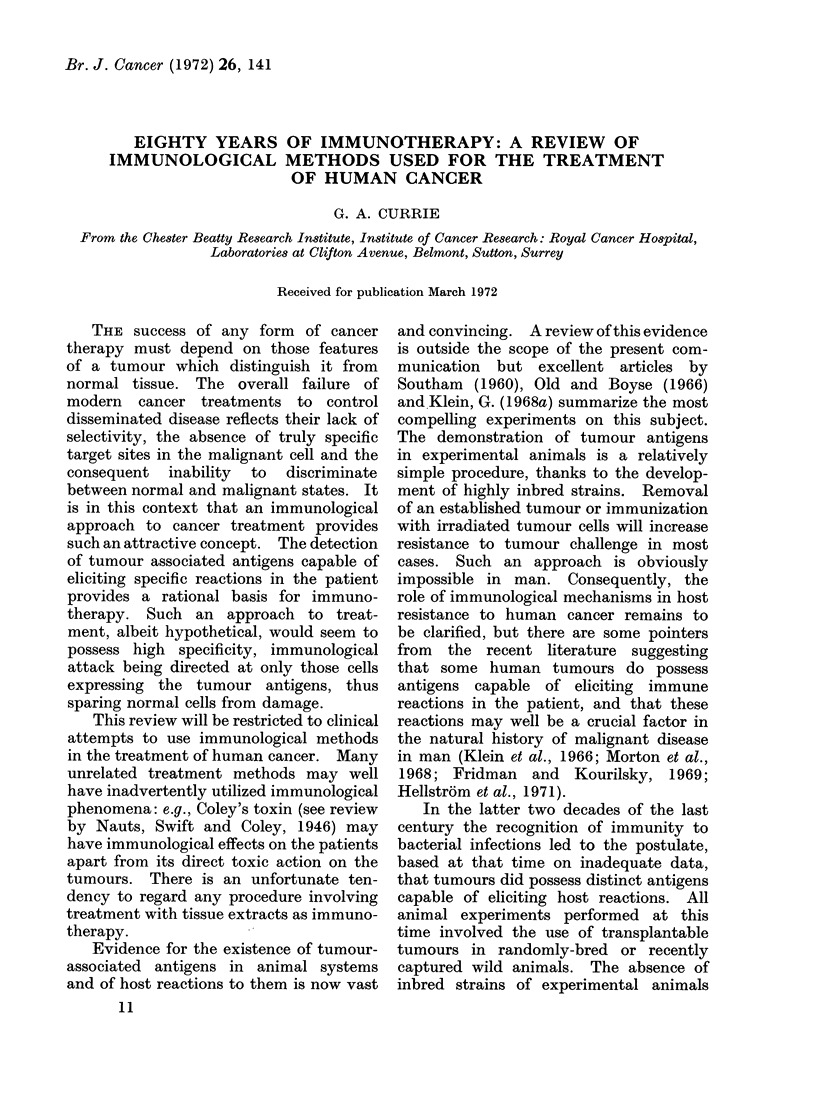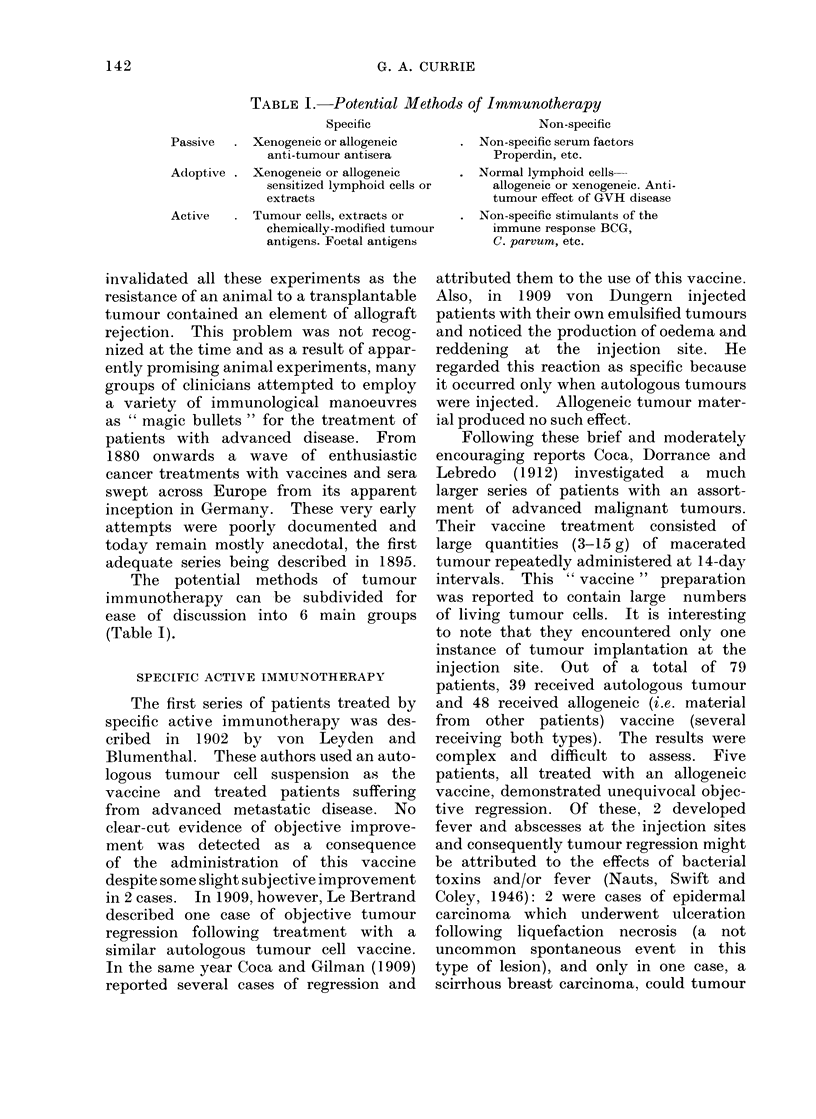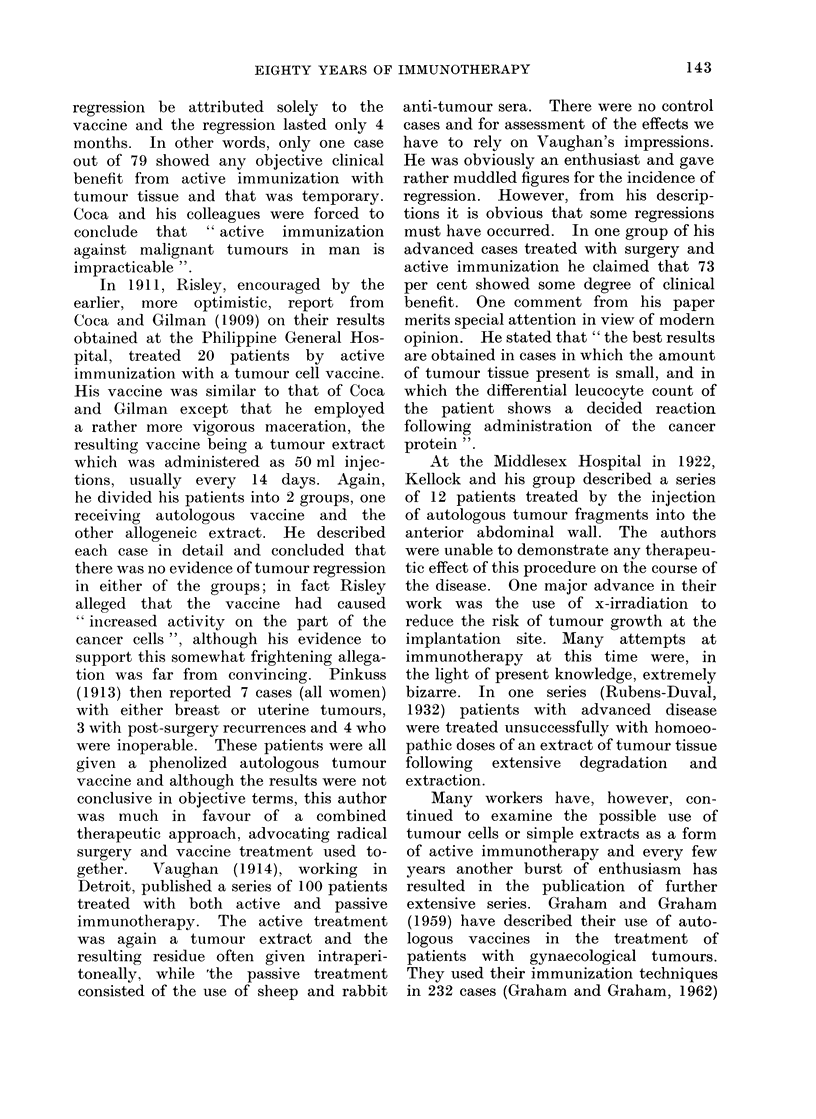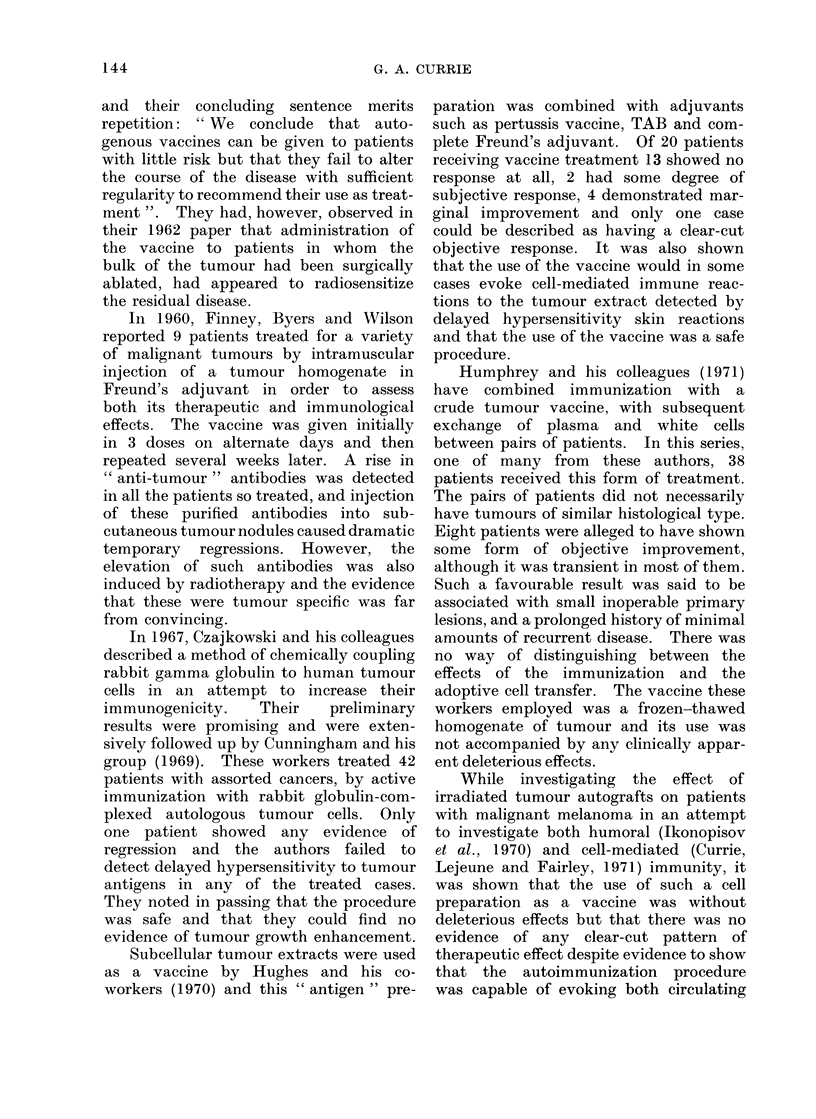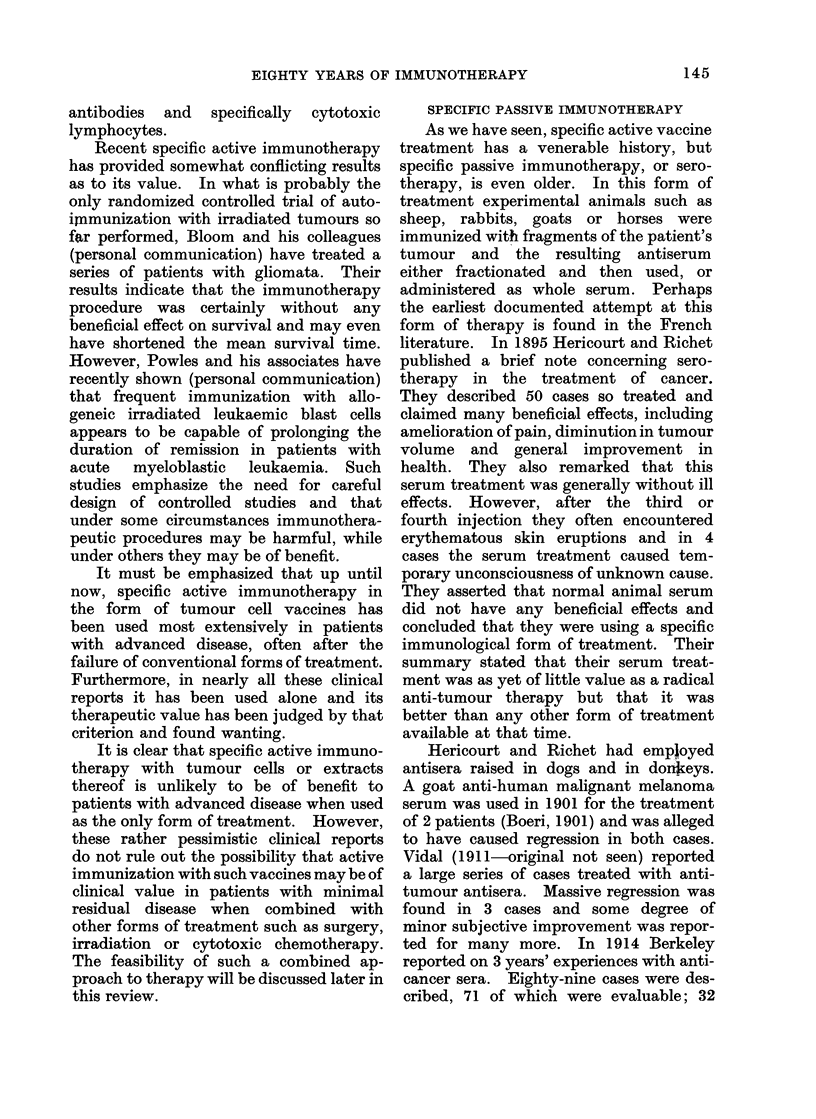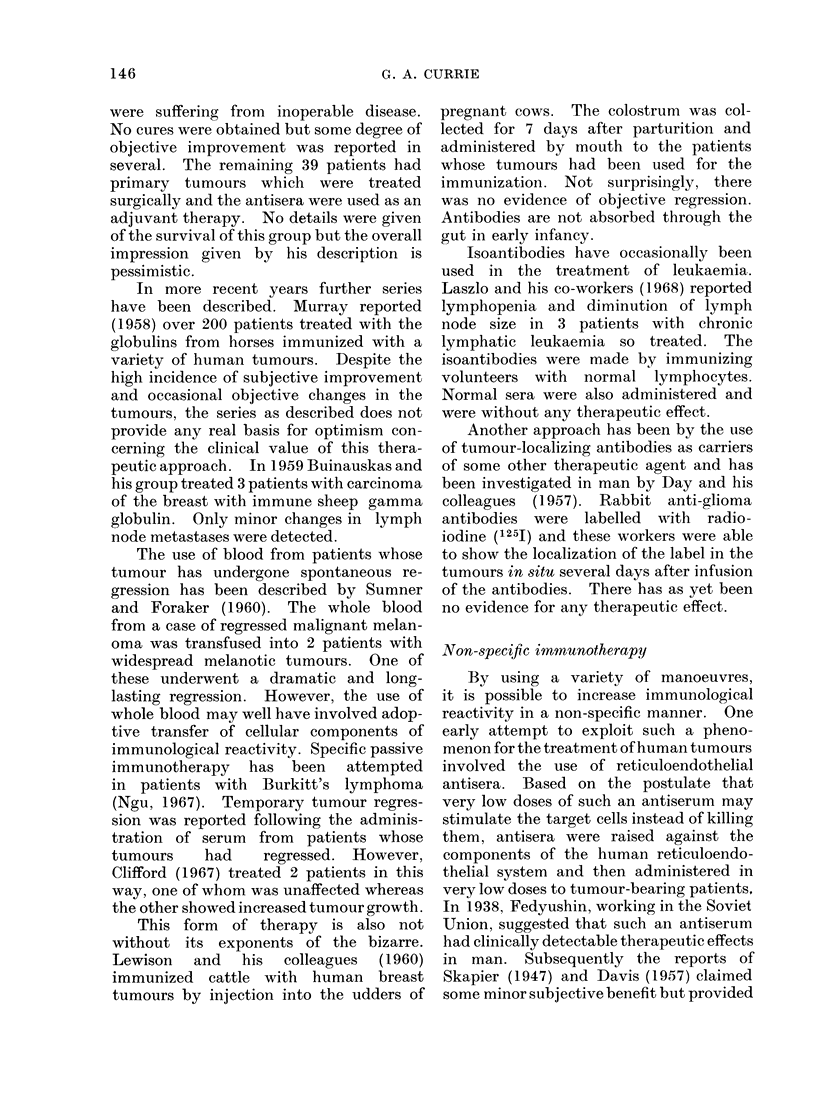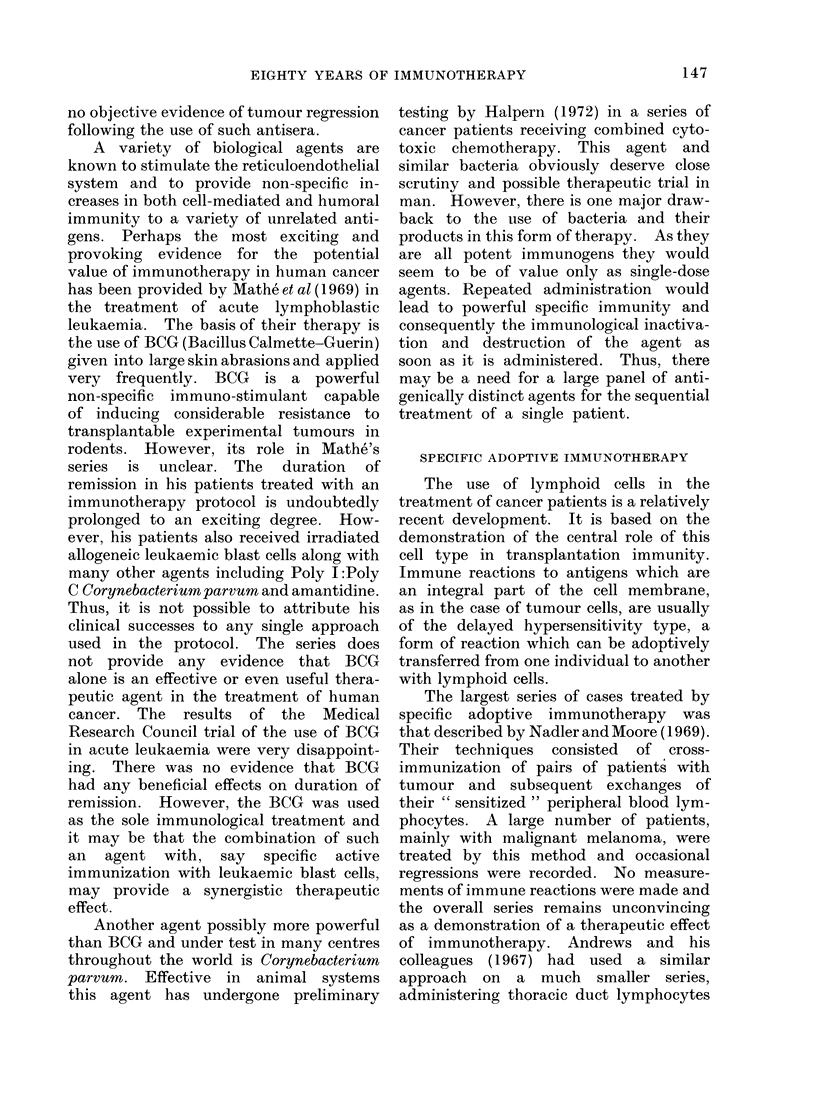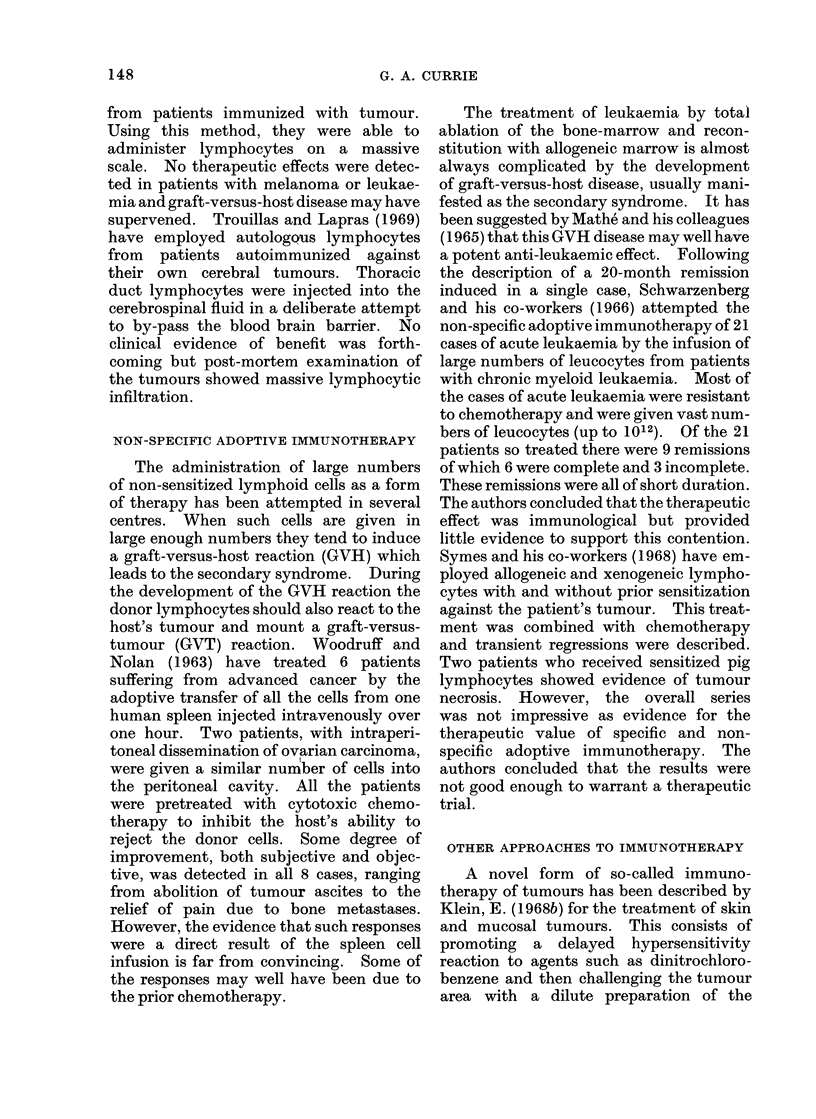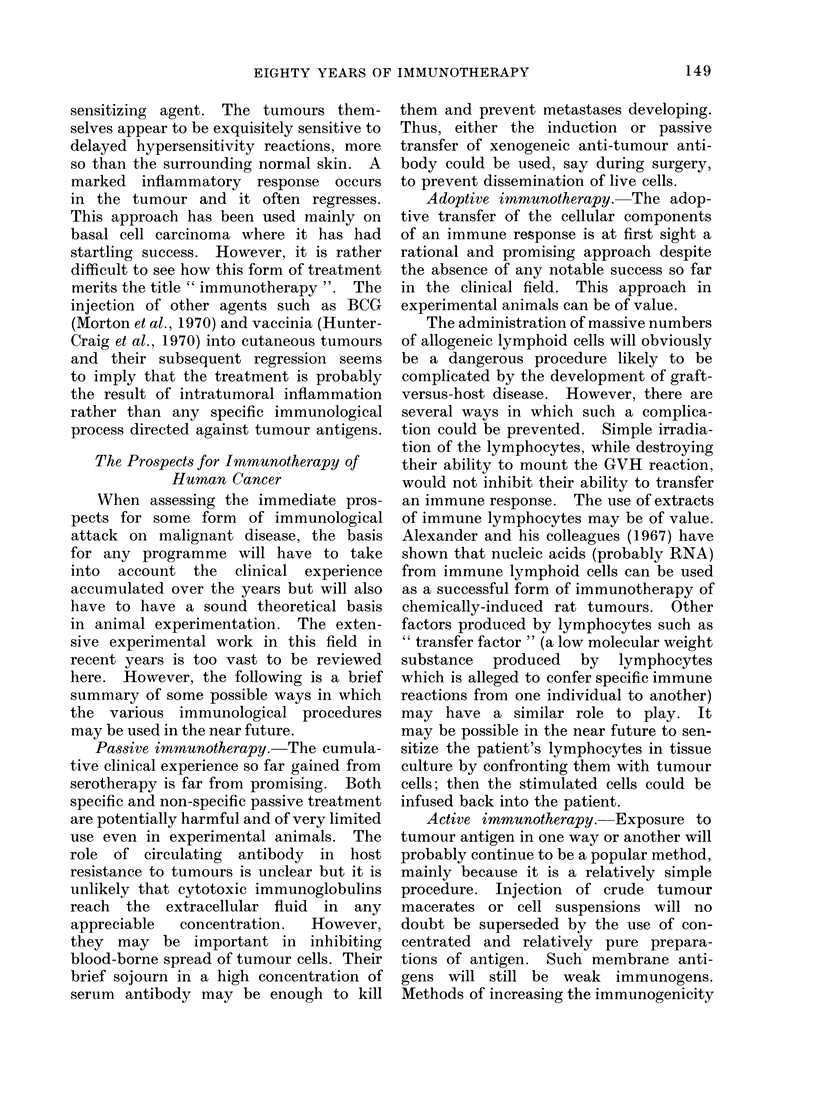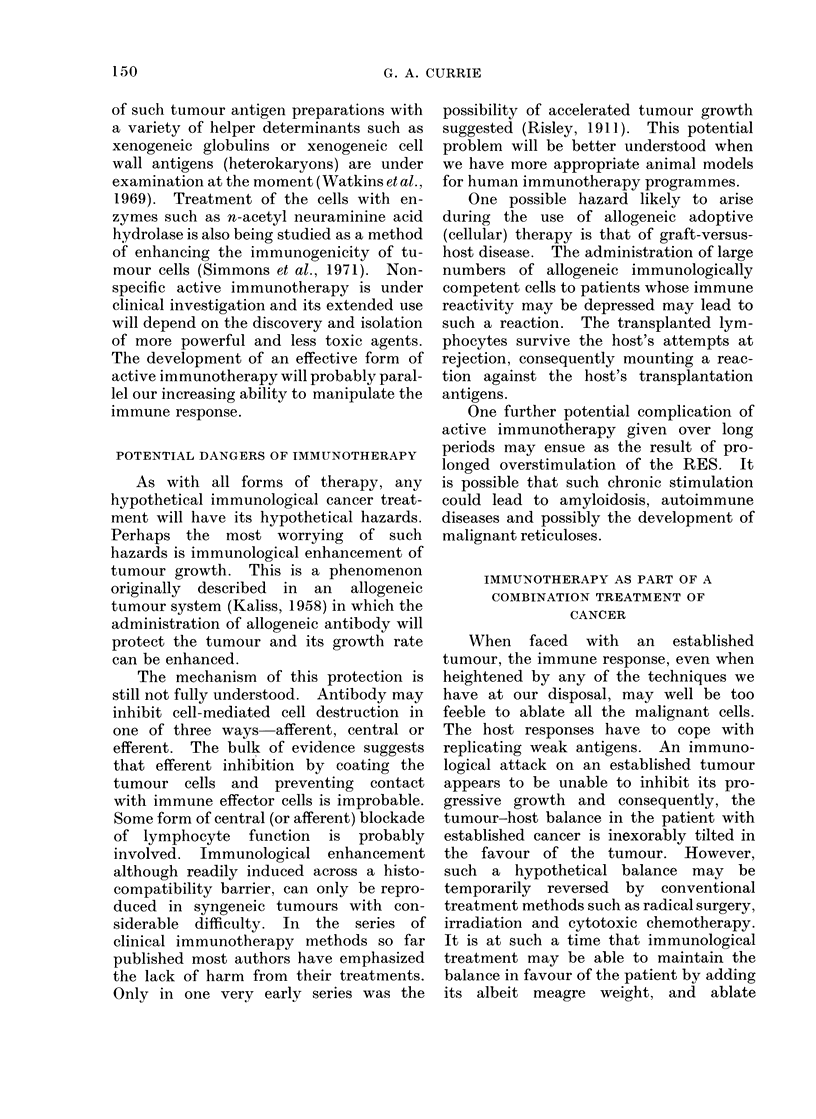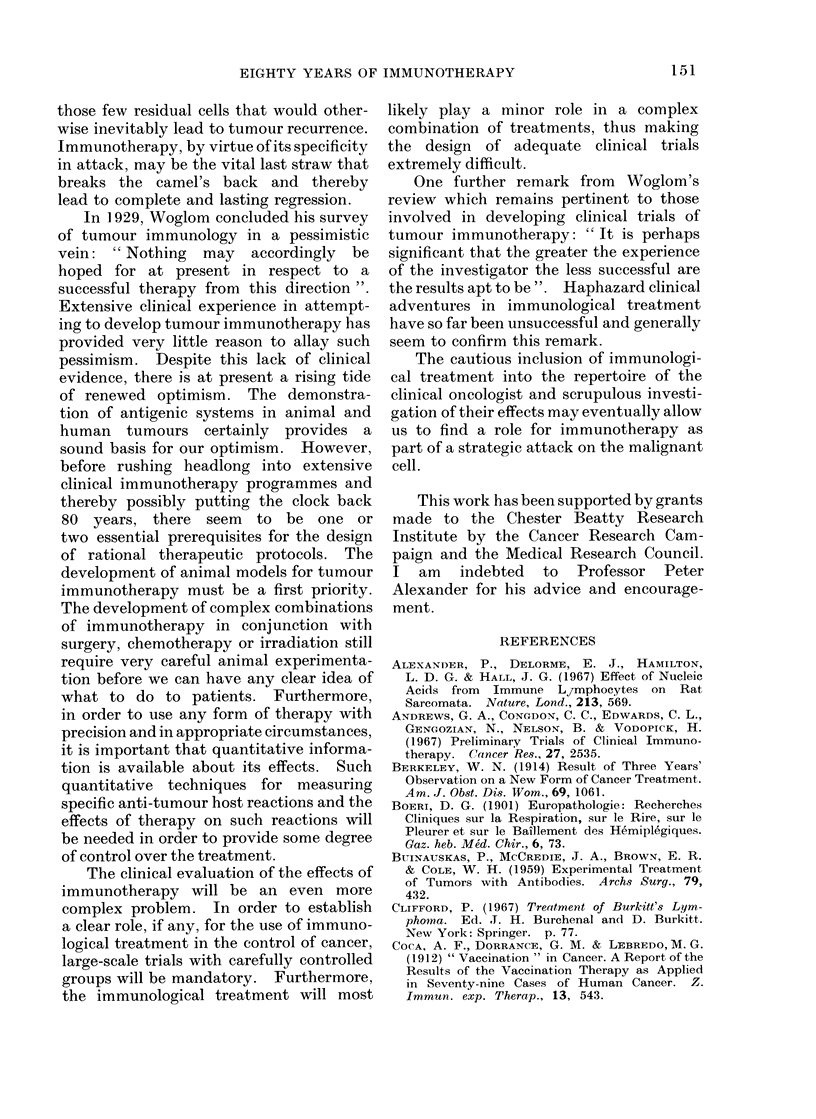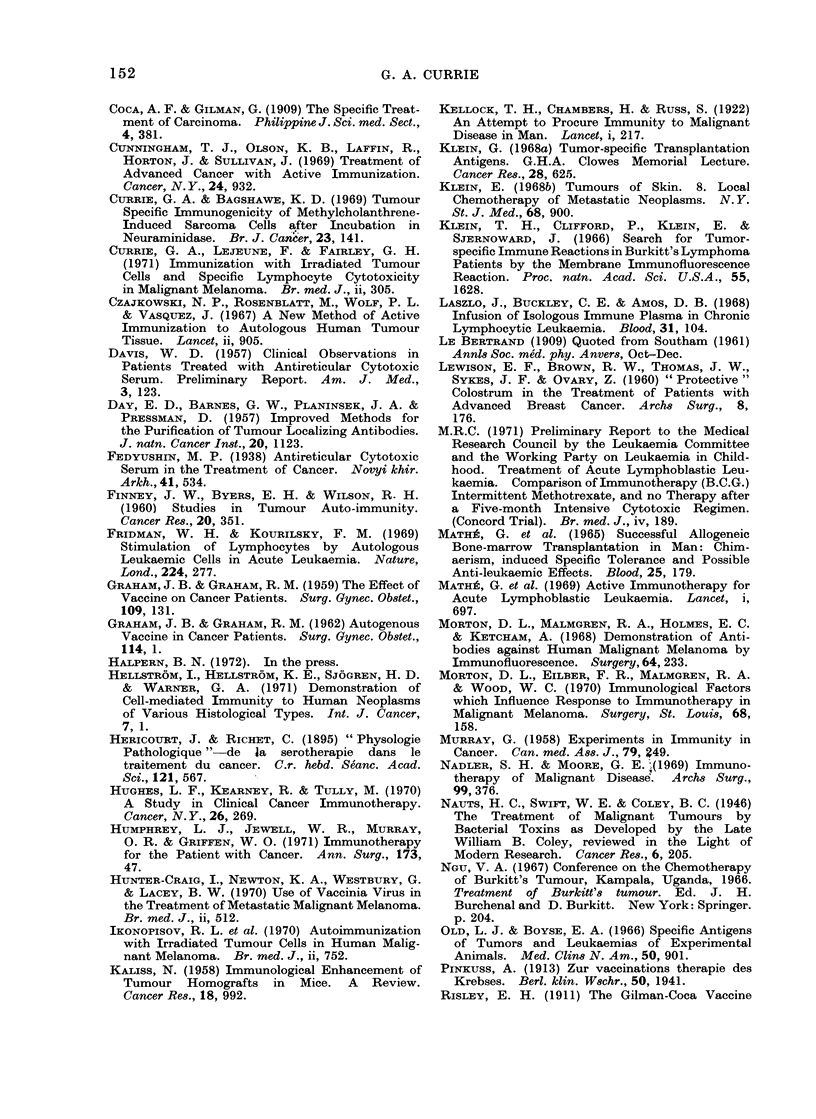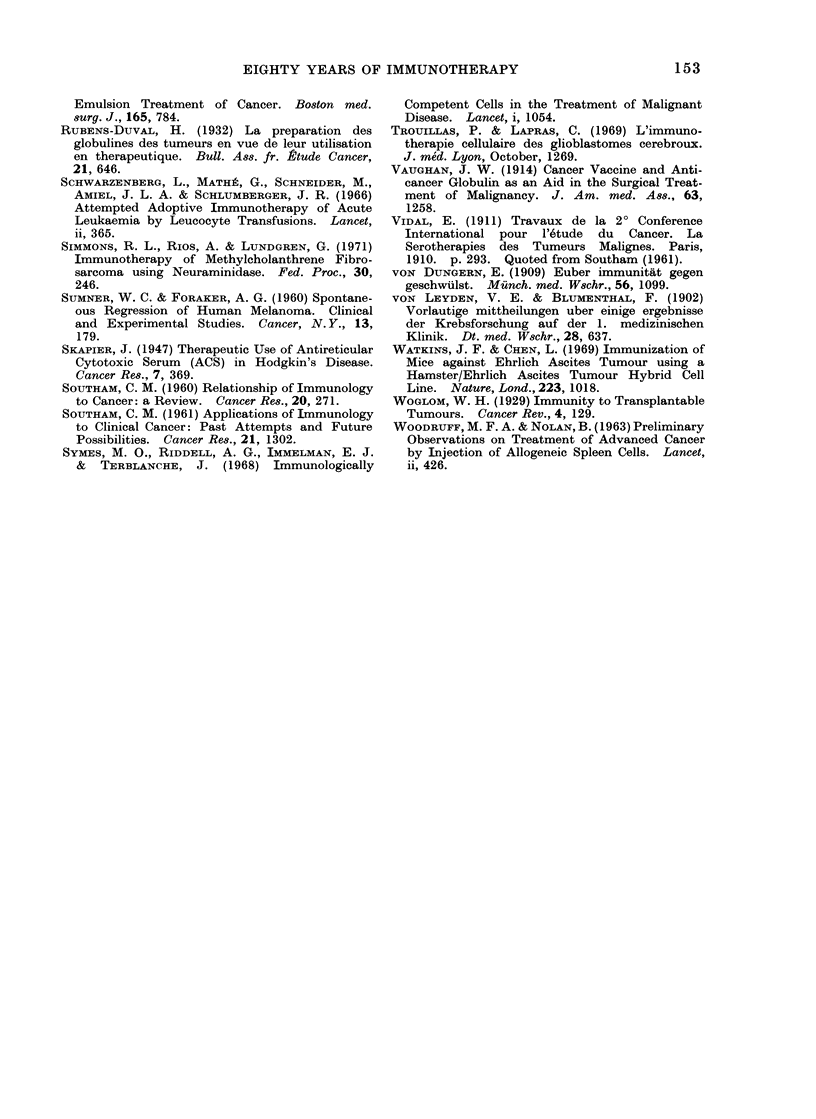# Eighty years of immunotherapy: a review of immunological methods used for the treatment of human cancer.

**DOI:** 10.1038/bjc.1972.21

**Published:** 1972-06

**Authors:** G. A. Currie


*Br. J. Cancer* (1972) **26**, 141

### EIGHTY YEARS OF IMMUNOTHERAPY: A REVIEW OF IMMUNOLOGICAL METHODS USED FOR THE TREATMENT OF HUMAN CANCER

G. A. CURRIE

*From the Chester Beatty Research Institute, Institute of Cancer Research: Royal Cancer Hospital, Laboratories at Clifton Avenue, Belmont, Sutton, Surrey*

Received for publication March 1972

THE success of any form of cancer therapy must depend on those features of a tumour which distinguish it from normal tissue. The overall failure of modern cancer treatments to control disseminated disease reflects their lack of selectivity, the absence of truly specific target sites in the malignant cell and the consequent inability to discriminate between normal and malignant states. It is in this context that an immunological approach to cancer treatment provides such an attractive concept. The detection of tumour associated antigens capable of eliciting specific reactions in the patient provides a rational basis for immunotherapy. Such an approach to treatment, albeit hypothetical, would seem to possess high specificity, immunological attack being directed at only those cells expressing the tumour antigens, thus sparing normal cells from damage.

This review will be restricted to clinical attempts to use immunological methods in the treatment of human cancer. Many unrelated treatment methods may well have inadvertently utilized immunological phenomena: *e.g.*, Coley's toxin (see review by Nauts, Swift and Coley, 1946) may have immunological effects on the patients apart from its direct toxic action on the tumours. There is an unfortunate tendency to regard any procedure involving treatment with tissue extracts as immunotherapy.

Evidence for the existence of tumour-associated antigens in animal systems and of host reactions to them is now vast and convincing. A review of this evidence is outside the scope of the present communication but excellent articles by Southam (1960), Old and Boyse (1966) and Klein, G. (1968*a*) summarize the most compelling experiments on this subject. The demonstration of tumour antigens in experimental animals is a relatively simple procedure, thanks to the development of highly inbred strains. Removal of an established tumour or immunization with irradiated tumour cells will increase resistance to tumour challenge in most cases. Such an approach is obviously impossible in man. Consequently, the role of immunological mechanisms in host resistance to human cancer remains to be clarified, but there are some pointers from the recent literature suggesting that some human tumours do possess antigens capable of eliciting immune reactions in the patient, and that these reactions may well be a crucial factor in the natural history of malignant disease in man (Klein *et al.*, 1966; Morton *et al.*, 1968; Fridman and Kourilsky, 1969; Hellström *et al.*, 1971).

In the latter two decades of the last century the recognition of immunity to bacterial infections led to the postulate, based at that time on inadequate data, that tumours did possess distinct antigens capable of eliciting host reactions. All animal experiments performed at this time involved the use of transplantable tumours in randomly-bred or recently captured wild animals. The absence of inbred strains of experimental animals

TABLE I.—*Potential Methods of Immunotherapy*

| | Specific | Non-specific |
|---|---|---|
| Passive | Xenogeneic or allogeneic anti-tumour antisera | Non-specific serum factors Properdin, etc. |
| Adoptive | Xenogeneic or allogeneic sensitized lymphoid cells or extracts | Normal lymphoid cells—allogeneic or xenogeneic. Anti-tumour effect of GVH disease |
| Active | Tumour cells, extracts or chemically-modified tumour antigens. Foetal antigens | Non-specific stimulants of the immune response BCG, *C. parvum*, etc. |

invalidated all these experiments as the resistance of an animal to a transplantable tumour contained an element of allograft rejection. This problem was not recognized at the time and as a result of apparently promising animal experiments, many groups of clinicians attempted to employ a variety of immunological manoeuvres as " magic bullets " for the treatment of patients with advanced disease. From 1880 onwards a wave of enthusiastic cancer treatments with vaccines and sera swept across Europe from its apparent inception in Germany. These very early attempts were poorly documented and today remain mostly anecdotal, the first adequate series being described in 1895.

The potential methods of tumour immunotherapy can be subdivided for ease of discussion into 6 main groups (Table I).

### SPECIFIC ACTIVE IMMUNOTHERAPY

The first series of patients treated by specific active immunotherapy was described in 1902 by von Leyden and Blumenthal. These authors used an autologous tumour cell suspension as the vaccine and treated patients suffering from advanced metastatic disease. No clear-cut evidence of objective improvement was detected as a consequence of the administration of this vaccine despite some slight subjective improvement in 2 cases. In 1909, however, Le Bertrand described one case of objective tumour regression following treatment with a similar autologous tumour cell vaccine. In the same year Coca and Gilman (1909) reported several cases of regression and attributed them to the use of this vaccine. Also, in 1909 von Dungern injected patients with their own emulsified tumours and noticed the production of oedema and reddening at the injection site. He regarded this reaction as specific because it occurred only when autologous tumours were injected. Allogeneic tumour material produced no such effect.

Following these brief and moderately encouraging reports Coca, Dorrance and Lebredo (1912) investigated a much larger series of patients with an assortment of advanced malignant tumours. Their vaccine treatment consisted of large quantities (3–15 g) of macerated tumour repeatedly administered at 14-day intervals. This " vaccine " preparation was reported to contain large numbers of living tumour cells. It is interesting to note that they encountered only one instance of tumour implantation at the injection site. Out of a total of 79 patients, 39 received autologous tumour and 48 received allogeneic (*i.e.* material from other patients) vaccine (several receiving both types). The results were complex and difficult to assess. Five patients, all treated with an allogeneic vaccine, demonstrated unequivocal objective regression. Of these, 2 developed fever and abscesses at the injection sites and consequently tumour regression might be attributed to the effects of bacterial toxins and/or fever (Nauts, Swift and Coley, 1946): 2 were cases of epidermal carcinoma which underwent ulceration following liquefaction necrosis (a not uncommon spontaneous event in this type of lesion), and only in one case, a scirrhous breast carcinoma, could tumour regression be attributed solely to the vaccine and the regression lasted only 4 months. In other words, only one case out of 79 showed any objective clinical benefit from active immunization with tumour tissue and that was temporary. Coca and his colleagues were forced to conclude that " active immunization against malignant tumours in man is impracticable ".

In 1911, Risley, encouraged by the earlier, more optimistic, report from Coca and Gilman (1909) on their results obtained at the Philippine General Hospital, treated 20 patients by active immunization with a tumour cell vaccine. His vaccine was similar to that of Coca and Gilman except that he employed a rather more vigorous maceration, the resulting vaccine being a tumour extract which was administered as 50 ml injections, usually every 14 days. Again, he divided his patients into 2 groups, one receiving autologous vaccine and the other allogeneic extract. He described each case in detail and concluded that there was no evidence of tumour regression in either of the groups; in fact Risley alleged that the vaccine had caused " increased activity on the part of the cancer cells ", although his evidence to support this somewhat frightening allegation was far from convincing. Pinkuss (1913) then reported 7 cases (all women) with either breast or uterine tumours, 3 with post-surgery recurrences and 4 who were inoperable. These patients were all given a phenolized autologous tumour vaccine and although the results were not conclusive in objective terms, this author was much in favour of a combined therapeutic approach, advocating radical surgery and vaccine treatment used together. Vaughan (1914), working in Detroit, published a series of 100 patients treated with both active and passive immunotherapy. The active treatment was again a tumour extract and the resulting residue often given intraperitoneally, while the passive treatment consisted of the use of sheep and rabbit anti-tumour sera. There were no control cases and for assessment of the effects we have to rely on Vaughan's impressions. He was obviously an enthusiast and gave rather muddled figures for the incidence of regression. However, from his descriptions it is obvious that some regressions must have occurred. In one group of his advanced cases treated with surgery and active immunization he claimed that 73 per cent showed some degree of clinical benefit. One comment from his paper merits special attention in view of modern opinion. He stated that " the best results are obtained in cases in which the amount of tumour tissue present is small, and in which the differential leucocyte count of the patient shows a decided reaction following administration of the cancer protein ".

At the Middlesex Hospital in 1922, Kellock and his group described a series of 12 patients treated by the injection of autologous tumour fragments into the anterior abdominal wall. The authors were unable to demonstrate any therapeutic effect of this procedure on the course of the disease. One major advance in their work was the use of x-irradiation to reduce the risk of tumour growth at the implantation site. Many attempts at immunotherapy at this time were, in the light of present knowledge, extremely bizarre. In one series (Rubens-Duval, 1932) patients with advanced disease were treated unsuccessfully with homoeopathic doses of an extract of tumour tissue following extensive degradation and extraction.

Many workers have, however, continued to examine the possible use of tumour cells or simple extracts as a form of active immunotherapy and every few years another burst of enthusiasm has resulted in the publication of further extensive series. Graham and Graham (1959) have described their use of autologous vaccines in the treatment of patients with gynaecological tumours. They used their immunization techniques in 232 cases (Graham and Graham, 1962)

and their concluding sentence merits repetition: " We conclude that autogenous vaccines can be given to patients with little risk but that they fail to alter the course of the disease with sufficient regularity to recommend their use as treatment ". They had, however, observed in their 1962 paper that administration of the vaccine to patients in whom the bulk of the tumour had been surgically ablated, had appeared to radiosensitize the residual disease.

In 1960, Finney, Byers and Wilson reported 9 patients treated for a variety of malignant tumours by intramuscular injection of a tumour homogenate in Freund's adjuvant in order to assess both its therapeutic and immunological effects. The vaccine was given initially in 3 doses on alternate days and then repeated several weeks later. A rise in " anti-tumour " antibodies was detected in all the patients so treated, and injection of these purified antibodies into subcutaneous tumour nodules caused dramatic temporary regressions. However, the elevation of such antibodies was also induced by radiotherapy and the evidence that these were tumour specific was far from convincing.

In 1967, Czajkowski and his colleagues described a method of chemically coupling rabbit gamma globulin to human tumour cells in an attempt to increase their immunogenicity. Their preliminary results were promising and were extensively followed up by Cunningham and his group (1969). These workers treated 42 patients with assorted cancers, by active immunization with rabbit globulin-complexed autologous tumour cells. Only one patient showed any evidence of regression and the authors failed to detect delayed hypersensitivity to tumour antigens in any of the treated cases. They noted in passing that the procedure was safe and that they could find no evidence of tumour growth enhancement.

Subcellular tumour extracts were used as a vaccine by Hughes and his coworkers (1970) and this " antigen " preparation was combined with adjuvants such as pertussis vaccine, TAB and complete Freund's adjuvant. Of 20 patients receiving vaccine treatment 13 showed no response at all, 2 had some degree of subjective response, 4 demonstrated marginal improvement and only one case could be described as having a clear-cut objective response. It was also shown that the use of the vaccine would in some cases evoke cell-mediated immune reactions to the tumour extract detected by delayed hypersensitivity skin reactions and that the use of the vaccine was a safe procedure.

Humphrey and his colleagues (1971) have combined immunization with a crude tumour vaccine, with subsequent exchange of plasma and white cells between pairs of patients. In this series, one of many from these authors, 38 patients received this form of treatment. The pairs of patients did not necessarily have tumours of similar histological type. Eight patients were alleged to have shown some form of objective improvement, although it was transient in most of them. Such a favourable result was said to be associated with small inoperable primary lesions, and a prolonged history of minimal amounts of recurrent disease. There was no way of distinguishing between the effects of the immunization and the adoptive cell transfer. The vaccine these workers employed was a frozen–thawed homogenate of tumour and its use was not accompanied by any clinically apparent deleterious effects.

While investigating the effect of irradiated tumour autografts on patients with malignant melanoma in an attempt to investigate both humoral (Ikonopisov *et al.*, 1970) and cell-mediated (Currie, Lejeune and Fairley, 1971) immunity, it was shown that the use of such a cell preparation as a vaccine was without deleterious effects but that there was no evidence of any clear-cut pattern of therapeutic effect despite evidence to show that the autoimmunization procedure was capable of evoking both circulating antibodies and specifically cytotoxic lymphocytes.

Recent specific active immunotherapy has provided somewhat conflicting results as to its value. In what is probably the only randomized controlled trial of autoimmunization with irradiated tumours so far performed, Bloom and his colleagues (personal communication) have treated a series of patients with gliomata. Their results indicate that the immunotherapy procedure was certainly without any beneficial effect on survival and may even have shortened the mean survival time. However, Powles and his associates have recently shown (personal communication) that frequent immunization with allogeneic irradiated leukaemic blast cells appears to be capable of prolonging the duration of remission in patients with acute myeloblastic leukaemia. Such studies emphasize the need for careful design of controlled studies and that under some circumstances immunotherapeutic procedures may be harmful, while under others they may be of benefit.

It must be emphasized that up until now, specific active immunotherapy in the form of tumour cell vaccines has been used most extensively in patients with advanced disease, often after the failure of conventional forms of treatment. Furthermore, in nearly all these clinical reports it has been used alone and its therapeutic value has been judged by that criterion and found wanting.

It is clear that specific active immunotherapy with tumour cells or extracts thereof is unlikely to be of benefit to patients with advanced disease when used as the only form of treatment. However, these rather pessimistic clinical reports do not rule out the possibility that active immunization with such vaccines may be of clinical value in patients with minimal residual disease when combined with other forms of treatment such as surgery, irradiation or cytotoxic chemotherapy. The feasibility of such a combined approach to therapy will be discussed later in this review.

### SPECIFIC PASSIVE IMMUNOTHERAPY

As we have seen, specific active vaccine treatment has a venerable history, but specific passive immunotherapy, or serotherapy, is even older. In this form of treatment experimental animals such as sheep, rabbits, goats or horses were immunized with fragments of the patient's tumour and the resulting antiserum either fractionated and then used, or administered as whole serum. Perhaps the earliest documented attempt at this form of therapy is found in the French literature. In 1895 Hericourt and Richet published a brief note concerning serotherapy in the treatment of cancer. They described 50 cases so treated and claimed many beneficial effects, including amelioration of pain, diminution in tumour volume and general improvement in health. They also remarked that this serum treatment was generally without ill effects. However, after the third or fourth injection they often encountered erythematous skin eruptions and in 4 cases the serum treatment caused temporary unconsciousness of unknown cause. They asserted that normal animal serum did not have any beneficial effects and concluded that they were using a specific immunological form of treatment. Their summary stated that their serum treatment was as yet of little value as a radical anti-tumour therapy but that it was better than any other form of treatment available at that time.

Hericourt and Richet had employed antisera raised in dogs and in donkeys. A goat anti-human malignant melanoma serum was used in 1901 for the treatment of 2 patients (Boeri, 1901) and was alleged to have caused regression in both cases. Vidal (1911—original not seen) reported a large series of cases treated with anti-tumour antisera. Massive regression was found in 3 cases and some degree of minor subjective improvement was reported for many more. In 1914 Berkeley reported on 3 years' experiences with anti-cancer sera. Eighty-nine cases were described, 71 of which were evaluable; 32

were suffering from inoperable disease. No cures were obtained but some degree of objective improvement was reported in several. The remaining 39 patients had primary tumours which were treated surgically and the antisera were used as an adjuvant therapy. No details were given of the survival of this group but the overall impression given by his description is pessimistic.

In more recent years further series have been described. Murray reported (1958) over 200 patients treated with the globulins from horses immunized with a variety of human tumours. Despite the high incidence of subjective improvement and occasional objective changes in the tumours, the series as described does not provide any real basis for optimism concerning the clinical value of this therapeutic approach. In 1959 Buinauskas and his group treated 3 patients with carcinoma of the breast with immune sheep gamma globulin. Only minor changes in lymph node metastases were detected.

The use of blood from patients whose tumour has undergone spontaneous regression has been described by Sumner and Foraker (1960). The whole blood from a case of regressed malignant melanoma was transfused into 2 patients with widespread melanotic tumours. One of these underwent a dramatic and long-lasting regression. However, the use of whole blood may well have involved adoptive transfer of cellular components of immunological reactivity. Specific passive immunotherapy has been attempted in patients with Burkitt's lymphoma (Ngu, 1967). Temporary tumour regression was reported following the administration of serum from patients whose tumours had regressed. However, Clifford (1967) treated 2 patients in this way, one of whom was unaffected whereas the other showed increased tumour growth.

This form of therapy is also not without its exponents of the bizarre. Lewison and his colleagues (1960) immunized cattle with human breast tumours by injection into the udders of pregnant cows. The colostrum was collected for 7 days after parturition and administered by mouth to the patients whose tumours had been used for the immunization. Not surprisingly, there was no evidence of objective regression. Antibodies are not absorbed through the gut in early infancy.

Isoantibodies have occasionally been used in the treatment of leukaemia. Laszlo and his co-workers (1968) reported lymphopenia and diminution of lymph node size in 3 patients with chronic lymphatic leukaemia so treated. The isoantibodies were made by immunizing volunteers with normal lymphocytes. Normal sera were also administered and were without any therapeutic effect.

Another approach has been by the use of tumour-localizing antibodies as carriers of some other therapeutic agent and has been investigated in man by Day and his colleagues (1957). Rabbit anti-glioma antibodies were labelled with radio-iodine ($^{125}I$) and these workers were able to show the localization of the label in the tumours *in situ* several days after infusion of the antibodies. There has as yet been no evidence for any therapeutic effect.

### *Non-specific immunotherapy*

By using a variety of manoeuvres, it is possible to increase immunological reactivity in a non-specific manner. One early attempt to exploit such a phenomenon for the treatment of human tumours involved the use of reticuloendothelial antisera. Based on the postulate that very low doses of such an antiserum may stimulate the target cells instead of killing them, antisera were raised against the components of the human reticuloendothelial system and then administered in very low doses to tumour-bearing patients. In 1938, Fedyushin, working in the Soviet Union, suggested that such an antiserum had clinically detectable therapeutic effects in man. Subsequently the reports of Skapier (1947) and Davis (1957) claimed some minor subjective benefit but provided no objective evidence of tumour regression following the use of such antisera.

A variety of biological agents are known to stimulate the reticuloendothelial system and to provide non-specific increases in both cell-mediated and humoral immunity to a variety of unrelated antigens. Perhaps the most exciting and provoking evidence for the potential value of immunotherapy in human cancer has been provided by Mathé *et al* (1969) in the treatment of acute lymphoblastic leukaemia. The basis of their therapy is the use of BCG (Bacillus Calmette–Guerin) given into large skin abrasions and applied very frequently. BCG is a powerful non-specific immuno-stimulant capable of inducing considerable resistance to transplantable experimental tumours in rodents. However, its role in Mathé's series is unclear. The duration of remission in his patients treated with an immunotherapy protocol is undoubtedly prolonged to an exciting degree. However, his patients also received irradiated allogeneic leukaemic blast cells along with many other agents including Poly I:Poly C *Corynebacterium parvum* and amantidine. Thus, it is not possible to attribute his clinical successes to any single approach used in the protocol. The series does not provide any evidence that BCG alone is an effective or even useful therapeutic agent in the treatment of human cancer. The results of the Medical Research Council trial of the use of BCG in acute leukaemia were very disappointing. There was no evidence that BCG had any beneficial effects on duration of remission. However, the BCG was used as the sole immunological treatment and it may be that the combination of such an agent with, say specific active immunization with leukaemic blast cells, may provide a synergistic therapeutic effect.

Another agent possibly more powerful than BCG and under test in many centres throughout the world is *Corynebacterium parvum.* Effective in animal systems this agent has undergone preliminary testing by Halpern (1972) in a series of cancer patients receiving combined cytotoxic chemotherapy. This agent and similar bacteria obviously deserve close scrutiny and possible therapeutic trial in man. However, there is one major drawback to the use of bacteria and their products in this form of therapy. As they are all potent immunogens they would seem to be of value only as single-dose agents. Repeated administration would lead to powerful specific immunity and consequently the immunological inactivation and destruction of the agent as soon as it is administered. Thus, there may be a need for a large panel of antigenically distinct agents for the sequential treatment of a single patient.

### SPECIFIC ADOPTIVE IMMUNOTHERAPY

The use of lymphoid cells in the treatment of cancer patients is a relatively recent development. It is based on the demonstration of the central role of this cell type in transplantation immunity. Immune reactions to antigens which are an integral part of the cell membrane, as in the case of tumour cells, are usually of the delayed hypersensitivity type, a form of reaction which can be adoptively transferred from one individual to another with lymphoid cells.

The largest series of cases treated by specific adoptive immunotherapy was that described by Nadler and Moore (1969). Their techniques consisted of cross-immunization of pairs of patients with tumour and subsequent exchanges of their " sensitized " peripheral blood lymphocytes. A large number of patients, mainly with malignant melanoma, were treated by this method and occasional regressions were recorded. No measurements of immune reactions were made and the overall series remains unconvincing as a demonstration of a therapeutic effect of immunotherapy. Andrews and his colleagues (1967) had used a similar approach on a much smaller series, administering thoracic duct lymphocytes from patients immunized with tumour. Using this method, they were able to administer lymphocytes on a massive scale. No therapeutic effects were detected in patients with melanoma or leukaemia and graft-versus-host disease may have supervened. Trouillas and Lapras (1969) have employed autologous lymphocytes from patients autoimmunized against their own cerebral tumours. Thoracic duct lymphocytes were injected into the cerebrospinal fluid in a deliberate attempt to by-pass the blood brain barrier. No clinical evidence of benefit was forthcoming but post-mortem examination of the tumours showed massive lymphocytic infiltration.

### NON-SPECIFIC ADOPTIVE IMMUNOTHERAPY

The administration of large numbers of non-sensitized lymphoid cells as a form of therapy has been attempted in several centres. When such cells are given in large enough numbers they tend to induce a graft-versus-host reaction (GVH) which leads to the secondary syndrome. During the development of the GVH reaction the donor lymphocytes should also react to the host's tumour and mount a graft-versus-tumour (GVT) reaction. Woodruff and Nolan (1963) have treated 6 patients suffering from advanced cancer by the adoptive transfer of all the cells from one human spleen injected intravenously over one hour. Two patients, with intraperitoneal dissemination of ovarian carcinoma, were given a similar number of cells into the peritoneal cavity. All the patients were pretreated with cytotoxic chemotherapy to inhibit the host's ability to reject the donor cells. Some degree of improvement, both subjective and objective, was detected in all 8 cases, ranging from abolition of tumour ascites to the relief of pain due to bone metastases. However, the evidence that such responses were a direct result of the spleen cell infusion is far from convincing. Some of the responses may well have been due to the prior chemotherapy.

The treatment of leukaemia by total ablation of the bone-marrow and reconstitution with allogeneic marrow is almost always complicated by the development of graft-versus-host disease, usually manifested as the secondary syndrome. It has been suggested by Mathé and his colleagues (1965) that this GVH disease may well have a potent anti-leukaemic effect. Following the description of a 20-month remission induced in a single case, Schwarzenberg and his co-workers (1966) attempted the non-specific adoptive immunotherapy of 21 cases of acute leukaemia by the infusion of large numbers of leucocytes from patients with chronic myeloid leukaemia. Most of the cases of acute leukaemia were resistant to chemotherapy and were given vast numbers of leucocytes (up to $10^{12}$). Of the 21 patients so treated there were 9 remissions of which 6 were complete and 3 incomplete. These remissions were all of short duration. The authors concluded that the therapeutic effect was immunological but provided little evidence to support this contention. Symes and his co-workers (1968) have employed allogeneic and xenogeneic lymphocytes with and without prior sensitization against the patient's tumour. This treatment was combined with chemotherapy and transient regressions were described. Two patients who received sensitized pig lymphocytes showed evidence of tumour necrosis. However, the overall series was not impressive as evidence for the therapeutic value of specific and non-specific adoptive immunotherapy. The authors concluded that the results were not good enough to warrant a therapeutic trial.

### OTHER APPROACHES TO IMMUNOTHERAPY

A novel form of so-called immunotherapy of tumours has been described by Klein, E. (1968*b*) for the treatment of skin and mucosal tumours. This consists of promoting a delayed hypersensitivity reaction to agents such as dinitrochlorobenzene and then challenging the tumour area with a dilute preparation of the sensitizing agent. The tumours themselves appear to be exquisitely sensitive to delayed hypersensitivity reactions, more so than the surrounding normal skin. A marked inflammatory response occurs in the tumour and it often regresses. This approach has been used mainly on basal cell carcinoma where it has had startling success. However, it is rather difficult to see how this form of treatment merits the title " immunotherapy ". The injection of other agents such as BCG (Morton *et al.*, 1970) and vaccinia (Hunter-Craig *et al.*, 1970) into cutaneous tumours and their subsequent regression seems to imply that the treatment is probably the result of intratumoral inflammation rather than any specific immunological process directed against tumour antigens.

### *The Prospects for Immunotherapy of Human Cancer*

When assessing the immediate prospects for some form of immunological attack on malignant disease, the basis for any programme will have to take into account the clinical experience accumulated over the years but will also have to have a sound theoretical basis in animal experimentation. The extensive experimental work in this field in recent years is too vast to be reviewed here. However, the following is a brief summary of some possible ways in which the various immunological procedures may be used in the near future.

*Passive immunotherapy.*—The cumulative clinical experience so far gained from serotherapy is far from promising. Both specific and non-specific passive treatment are potentially harmful and of very limited use even in experimental animals. The role of circulating antibody in host resistance to tumours is unclear but it is unlikely that cytotoxic immunoglobulins reach the extracellular fluid in any appreciable concentration. However, they may be important in inhibiting blood-borne spread of tumour cells. Their brief sojourn in a high concentration of serum antibody may be enough to kill them and prevent metastases developing. Thus, either the induction or passive transfer of xenogeneic anti-tumour antibody could be used, say during surgery, to prevent dissemination of live cells.

*Adoptive immunotherapy.*—The adoptive transfer of the cellular components of an immune response is at first sight a rational and promising approach despite the absence of any notable success so far in the clinical field. This approach in experimental animals can be of value.

The administration of massive numbers of allogeneic lymphoid cells will obviously be a dangerous procedure likely to be complicated by the development of graft-versus-host disease. However, there are several ways in which such a complication could be prevented. Simple irradiation of the lymphocytes, while destroying their ability to mount the GVH reaction, would not inhibit their ability to transfer an immune response. The use of extracts of immune lymphocytes may be of value. Alexander and his colleagues (1967) have shown that nucleic acids (probably RNA) from immune lymphoid cells can be used as a successful form of immunotherapy of chemically-induced rat tumours. Other factors produced by lymphocytes such as " transfer factor " (a low molecular weight substance produced by lymphocytes which is alleged to confer specific immune reactions from one individual to another) may have a similar role to play. It may be possible in the near future to sensitize the patient's lymphocytes in tissue culture by confronting them with tumour cells; then the stimulated cells could be infused back into the patient.

*Active immunotherapy.*—Exposure to tumour antigen in one way or another will probably continue to be a popular method, mainly because it is a relatively simple procedure. Injection of crude tumour macerates or cell suspensions will no doubt be superseded by the use of concentrated and relatively pure preparations of antigen. Such membrane antigens will still be weak immunogens. Methods of increasing the immunogenicity of such tumour antigen preparations with a variety of helper determinants such as xenogeneic globulins or xenogeneic cell wall antigens (heterokaryons) are under examination at the moment (Watkins *et al.*, 1969). Treatment of the cells with enzymes such as *n*-acetyl neuraminine acid hydrolase is also being studied as a method of enhancing the immunogenicity of tumour cells (Simmons *et al.*, 1971). Non-specific active immunotherapy is under clinical investigation and its extended use will depend on the discovery and isolation of more powerful and less toxic agents. The development of an effective form of active immunotherapy will probably parallel our increasing ability to manipulate the immune response.

### POTENTIAL DANGERS OF IMMUNOTHERAPY

As with all forms of therapy, any hypothetical immunological cancer treatment will have its hypothetical hazards. Perhaps the most worrying of such hazards is immunological enhancement of tumour growth. This is a phenomenon originally described in an allogeneic tumour system (Kaliss, 1958) in which the administration of allogeneic antibody will protect the tumour and its growth rate can be enhanced.

The mechanism of this protection is still not fully understood. Antibody may inhibit cell-mediated cell destruction in one of three ways—afferent, central or efferent. The bulk of evidence suggests that efferent inhibition by coating the tumour cells and preventing contact with immune effector cells is improbable. Some form of central (or afferent) blockade of lymphocyte function is probably involved. Immunological enhancement although readily induced across a histocompatibility barrier, can only be reproduced in syngeneic tumours with considerable difficulty. In the series of clinical immunotherapy methods so far published most authors have emphasized the lack of harm from their treatments. Only in one very early series was the possibility of accelerated tumour growth suggested (Risley, 1911). This potential problem will be better understood when we have more appropriate animal models for human immunotherapy programmes.

One possible hazard likely to arise during the use of allogeneic adoptive (cellular) therapy is that of graft-versus-host disease. The administration of large numbers of allogeneic immunologically competent cells to patients whose immune reactivity may be depressed may lead to such a reaction. The transplanted lymphocytes survive the host's attempts at rejection, consequently mounting a reaction against the host's transplantation antigens.

One further potential complication of active immunotherapy given over long periods may ensue as the result of prolonged overstimulation of the RES. It is possible that such chronic stimulation could lead to amyloidosis, autoimmune diseases and possibly the development of malignant reticuloses.

### IMMUNOTHERAPY AS PART OF A COMBINATION TREATMENT OF CANCER

When faced with an established tumour, the immune response, even when heightened by any of the techniques we have at our disposal, may well be too feeble to ablate all the malignant cells. The host responses have to cope with replicating weak antigens. An immunological attack on an established tumour appears to be unable to inhibit its progressive growth and consequently, the tumour–host balance in the patient with established cancer is inexorably tilted in the favour of the tumour. However, such a hypothetical balance may be temporarily reversed by conventional treatment methods such as radical surgery, irradiation and cytotoxic chemotherapy. It is at such a time that immunological treatment may be able to maintain the balance in favour of the patient by adding its albeit meagre weight, and ablate those few residual cells that would otherwise inevitably lead to tumour recurrence. Immunotherapy, by virtue of its specificity in attack, may be the vital last straw that breaks the camel's back and thereby lead to complete and lasting regression.

In 1929, Woglom concluded his survey of tumour immunology in a pessimistic vein: " Nothing may accordingly be hoped for at present in respect to a successful therapy from this direction ". Extensive clinical experience in attempting to develop tumour immunotherapy has provided very little reason to allay such pessimism. Despite this lack of clinical evidence, there is at present a rising tide of renewed optimism. The demonstration of antigenic systems in animal and human tumours certainly provides a sound basis for our optimism. However, before rushing headlong into extensive clinical immunotherapy programmes and thereby possibly putting the clock back 80 years, there seem to be one or two essential prerequisites for the design of rational therapeutic protocols. The development of animal models for tumour immunotherapy must be a first priority. The development of complex combinations of immunotherapy in conjunction with surgery, chemotherapy or irradiation still require very careful animal experimentation before we can have any clear idea of what to do to patients. Furthermore, in order to use any form of therapy with precision and in appropriate circumstances, it is important that quantitative information is available about its effects. Such quantitative techniques for measuring specific anti-tumour host reactions and the effects of therapy on such reactions will be needed in order to provide some degree of control over the treatment.

The clinical evaluation of the effects of immunotherapy will be an even more complex problem. In order to establish a clear role, if any, for the use of immunological treatment in the control of cancer, large-scale trials with carefully controlled groups will be mandatory. Furthermore, the immunological treatment will most likely play a minor role in a complex combination of treatments, thus making the design of adequate clinical trials extremely difficult.

One further remark from Woglom's review which remains pertinent to those involved in developing clinical trials of tumour immunotherapy: " It is perhaps significant that the greater the experience of the investigator the less successful are the results apt to be ". Haphazard clinical adventures in immunological treatment have so far been unsuccessful and generally seem to confirm this remark.

The cautious inclusion of immunological treatment into the repertoire of the clinical oncologist and scrupulous investigation of their effects may eventually allow us to find a role for immunotherapy as part of a strategic attack on the malignant cell.

This work has been supported by grants made to the Chester Beatty Research Institute by the Cancer Research Campaign and the Medical Research Council. I am indebted to Professor Peter Alexander for his advice and encouragement.

### REFERENCES

ALEXANDER, P., DELORME, E. J., HAMILTON, L. D. G. & HALL, J. G. (1967) Effect of Nucleic Acids from Immune Lymphocytes on Rat Sarcomata. *Nature, Lond.*, **213**, 569.

ANDREWS, G. A., CONGDON, C. C., EDWARDS, C. L., GENGOZIAN, N., NELSON, B. & VODOPICK, H. (1967) Preliminary Trials of Clinical Immunotherapy. *Cancer Res.*, **27**, 2535.

BERKELEY, W. N. (1914) Result of Three Years' Observation on a New Form of Cancer Treatment. *Am. J. Obst. Dis. Wom.*, **69**, 1061.

BOERI, D. G. (1901) Europathologie: Recherches Cliniques sur la Respiration, sur le Rire, sur le Pleurer et sur le Bâillement des Hémiplégiques. *Gaz. heb. Méd. Chir.*, **6**, 73.

BUINAUSKAS, P., MCCREDIE, J. A., BROWN, E. R. & COLE, W. H. (1959) Experimental Treatment of Tumors with Antibodies. *Archs Surg.*, **79**, 432.

CLIFFORD, P. (1967) *Treatment of Burkitt's Lymphoma.* Ed. J. H. Burchenal and D. Burkitt. New York: Springer. p. 77.

COCA, A. F., DORRANCE, G. M. & LEBREDO, M. G. (1912) " Vaccination " in Cancer. A Report of the Results of the Vaccination Therapy as Applied in Seventy-nine Cases of Human Cancer. *Z. Immun. exp. Therap.*, **13**, 543.

COCA, A. F. & GILMAN, G. (1909) The Specific Treatment of Carcinoma. *Philippine J. Sci. med. Sect.*, **4**, 381.

CUNNINGHAM, T. J., OLSON, K. B., LAFFIN, R., HORTON, J. & SULLIVAN, J. (1969) Treatment of Advanced Cancer with Active Immunization. *Cancer, N.Y.*, **24**, 932.

CURRIE, G. A. & BAGSHAWE, K. D. (1969) Tumour Specific Immunogenicity of Methylcholanthrene-Induced Sarcoma Cells after Incubation in Neuraminidase. *Br. J. Cancer*, **23**, 141.

CURRIE, G. A., LEJEUNE, F. & FAIRLEY, G. H. (1971) Immunization with Irradiated Tumour Cells and Specific Lymphocyte Cytotoxicity in Malignant Melanoma. *Br. med. J.*, ii, 305.

CZAJKOWSKI, N. P., ROSENBLATT, M., WOLF, P. L. & VASQUEZ, J. (1967) A New Method of Active Immunization to Autologous Human Tumour Tissue. *Lancet*, ii, 905.

DAVIS, W. D. (1957) Clinical Observations in Patients Treated with Antireticular Cytotoxic Serum. Preliminary Report. *Am. J. Med.*, **3**, 123.

DAY, E. D., BARNES, G. W., PLANINSEK, J. A. & PRESSMAN, D. (1957) Improved Methods for the Purification of Tumour Localizing Antibodies. *J. natn. Cancer Inst.*, **20**, 1123.

FEDYUSHIN, M. P. (1938) Antireticular Cytotoxic Serum in the Treatment of Cancer. *Novyi khir. Arkh.*, **41**, 534.

FINNEY, J. W., BYERS, E. H. & WILSON, R. H. (1960) Studies in Tumour Auto-immunity. *Cancer Res.*, **20**, 351.

FRIDMAN, W. H. & KOURILSKY, F. M. (1969) Stimulation of Lymphocytes by Autologous Leukaemic Cells in Acute Leukaemia. *Nature, Lond.*, **224**, 277.

GRAHAM, J. B. & GRAHAM, R. M. (1959) The Effect of Vaccine on Cancer Patients. *Surg. Gynec. Obstet.*, **109**, 131.

GRAHAM, J. B. & GRAHAM, R. M. (1962) Autogenous Vaccine in Cancer Patients. *Surg. Gynec. Obstet.*, **114**, 1.

HALPERN, B. N. (1972). In the press.

HELLSTRÖM, I., HELLSTRÖM, K. E., SJÖGREN, H. D. & WARNER, G. A. (1971) Demonstration of Cell-mediated Immunity to Human Neoplasms of Various Histological Types. *Int. J. Cancer*, **7**, 1.

HERICOURT, J. & RICHET, C. (1895) " Physologie Pathologique "—de la serotherapie dans le traitement du cancer. *C.r. hebd. Séanc. Acad. Sci.*, **121**, 567.

HUGHES, L. F., KEARNEY, R. & TULLY, M. (1970) A Study in Clinical Cancer Immunotherapy. *Cancer, N.Y.*, **26**, 269.

HUMPHREY, L. J., JEWELL, W. R., MURRAY, O. R. & GRIFFEN, W. O. (1971) Immunotherapy for the Patient with Cancer. *Ann. Surg.*, **173**, 47.

HUNTER-CRAIG, I., NEWTON, K. A., WESTBURY, G. & LACEY, B. W. (1970) Use of Vaccinia Virus in the Treatment of Metastatic Malignant Melanoma. *Br. med. J.*, ii, 512.

IKONOPISOV, R. L. *et al.* (1970) Autoimmunization with Irradiated Tumour Cells in Human Malignant Melanoma. *Br. med. J.*, ii, 752.

KALISS, N. (1958) Immunological Enhancement of Tumour Homografts in Mice. A Review. *Cancer Res.*, **18**, 992.

KELLOCK, T. H., CHAMBERS, H. & RUSS, S. (1922) An Attempt to Procure Immunity to Malignant Disease in Man. *Lancet*, i, 217.

KLEIN, G. (1968*a*) Tumor-specific Transplantation Antigens. G.H.A. Clowes Memorial Lecture. *Cancer Res.*, **28**, 625.

KLEIN, E. (1968*b*) Tumours of Skin. 8. Local Chemotherapy of Metastatic Neoplasms. *N.Y. St. J. Med.*, **68**, 900.

KLEIN, T. H., CLIFFORD, P., KLEIN, E. & SJERNOWARD, J. (1966) Search for Tumor-specific Immune Reactions in Burkitt's Lymphoma Patients by the Membrane Immunofluorescence Reaction. *Proc. natn. Acad. Sci. U.S.A.*, **55**, 1628.

LASZLO, J., BUCKLEY, C. E. & AMOS, D. B. (1968) Infusion of Isologous Immune Plasma in Chronic Lymphocytic Leukaemia. *Blood*, **31**, 104.

LE BERTRAND (1909) Quoted from Southam (1961) *Annls Soc. méd. phy. Anvers*, Oct–Dec.

LEWISON, E. F., BROWN, R. W., THOMAS, J. W., SYKES, J. F. & OVARY, Z. (1960) " Protective " Colostrum in the Treatment of Patients with Advanced Breast Cancer. *Archs Surg.*, **8**, 176.

M.R.C. (1971) Preliminary Report to the Medical Research Council by the Leukaemia Committee and the Working Party on Leukaemia in Childhood. Treatment of Acute Lymphoblastic Leukaemia. Comparison of Immunotherapy (B.C.G.) Intermittent Methotrexate, and no Therapy after a Five-month Intensive Cytotoxic Regimen. (Concord Trial). *Br. med. J.*, iv, 189.

MATHÉ, G. *et al.* (1965) Successful Allogeneic Bone-marrow Transplantation in Man: Chimaerism, induced Specific Tolerance and Possible Anti-leukaemic Effects. *Blood*, **25**, 179.

MATHÉ, G. *et al.* (1969) Active Immunotherapy for Acute Lymphoblastic Leukaemia. *Lancet*, i, 697.

MORTON, D. L., MALMGREN, R. A., HOLMES, E. C. & KETCHAM, A. (1968) Demonstration of Antibodies against Human Malignant Melanoma by Immunofluorescence. *Surgery*, **64**, 233.

MORTON, D. L., EILBER, F. R., MALMGREN, R. A. & WOOD, W. C. (1970) Immunological Factors which Influence Response to Immunotherapy in Malignant Melanoma. *Surgery, St. Louis*, **68**, 158.

MURRAY, G. (1958) Experiments in Immunity in Cancer. *Can. med. Ass. J.*, **79**, 249.

NADLER, S. H. & MOORE, G. E. (1969) Immunotherapy of Malignant Disease. *Archs Surg.*, **99**, 376.

NAUTS, H. C., SWIFT, W. E. & COLEY, B. C. (1946) The Treatment of Malignant Tumours by Bacterial Toxins as Developed by the Late William B. Coley, reviewed in the Light of Modern Research. *Cancer Res.*, **6**, 205.

NGU, V. A. (1967) Conference on the Chemotherapy of Burkitt's Tumour, Kampala, Uganda, 1966. *Treatment of Burkitt's tumour.* Ed. J. H. Burchenal and D. Burkitt. New York: Springer. p. 204.

OLD, L. J. & BOYSE, E. A. (1966) Specific Antigens of Tumors and Leukaemias of Experimental Animals. *Med. Clins N. Am.*, **50**, 901.

PINKUSS, A. (1913) Zur vaccinations therapie des Krebses. *Berl. klin. Wschr.*, **50**, 1941.

RISLEY, E. H. (1911) The Gilman-Coca Vaccine

Emulsion Treatment of Cancer. *Boston med. surg. J.*, **165**, 784.

RUBENS-DUVAL, H. (1932) La preparation des globulines des tumeurs en vue de leur utilisation en therapeutique. *Bull. Ass. fr. Étude Cancer*, **21**, 646.

SCHWARZENBERG, L., MATHÉ, G., SCHNEIDER, M., AMIEL, J. L. A. & SCHLUMBERGER, J. R. (1966) Attempted Adoptive Immunotherapy of Acute Leukaemia by Leucocyte Transfusions. *Lancet*, ii, 365.

SIMMONS, R. L., RIOS, A. & LUNDGREN, G. (1971) Immunotherapy of Methylcholanthrene Fibrosarcoma using Neuraminidase. *Fed. Proc.*, **30**, 246.

SUMNER, W. C. & FORAKER, A. G. (1960) Spontaneous Regression of Human Melanoma. Clinical and Experimental Studies. *Cancer, N.Y.*, **13**, 179.

SKAPIER, J. (1947) Therapeutic Use of Antireticular Cytotoxic Serum (ACS) in Hodgkin's Disease. *Cancer Res.*, **7**, 369.

SOUTHAM, C. M. (1960) Relationship of Immunology to Cancer: a Review. *Cancer Res.*, **20**, 271.

SOUTHAM, C. M. (1961) Applications of Immunology to Clinical Cancer: Past Attempts and Future Possibilities. *Cancer Res.*, **21**, 1302.

SYMES, M. O., RIDDELL, A. G., IMMELMAN, E. J. & TERBLANCHE, J. (1968) Immunologically Competent Cells in the Treatment of Malignant Disease. *Lancet*, i, 1054.

TROUILLAS, P. & LAPRAS, C. (1969) L'immunotherapie cellulaire des glioblastomes cerebroux. *J. méd. Lyon*, October, 1269.

VAUGHAN, J. W. (1914) Cancer Vaccine and Anticancer Globulin as an Aid in the Surgical Treatment of Malignancy. *J. Am. med. Ass.*, **63**, 1258.

VIDAL, E. (1911) Travaux de la 2° Conference International pour l'étude du Cancer. La Serotherapies des Tumeurs Malignes. Paris, 1910. p. 293. Quoted from Southam (1961).

VON DUNGERN, E. (1909) Euber immunität gegen geschwülst. *Münch. med. Wschr.*, **56**, 1099.

VON LEYDEN, V. E. & BLUMENTHAL, F. (1902) Vorlautige mittheilungen uber einige ergebnisse der Krebsforschung auf der 1. medizinischen Klinik. *Dt. med. Wschr.*, **28**, 637.

WATKINS, J. F. & CHEN, L. (1969) Immunization of Mice against Ehrlich Ascites Tumour using a Hamster/Ehrlich Ascites Tumour Hybrid Cell Line. *Nature, Lond.*, **223**, 1018.

WOGLOM, W. H. (1929) Immunity to Transplantable Tumours. *Cancer Rev.*, **4**, 129.

WOODRUFF, M. F. A. & NOLAN, B. (1963) Preliminary Observations on Treatment of Advanced Cancer by Injection of Allogeneic Spleen Cells. *Lancet*, ii, 426.